# A Nanomicellar Prodrug Carrier Based on Ibuprofen-Conjugated Polymer for Co-delivery of Doxorubicin

**DOI:** 10.3389/fphar.2018.00781

**Published:** 2018-08-14

**Authors:** Zuojun Li, Jingjing Sun, Yixian Huang, Yanhua Liu, Jieni Xu, Yichao Chen, Lei Liang, Jiang Li, Qiongfeng Liao, Song Li, Kechao Zhou

**Affiliations:** ^1^Department of Pharmacy, The Third Xiangya Hospital of Central South University, Changsha, China; ^2^State Key Laboratory of Powder Metallurgy, Department of Pharmaceutical Sciences, School of Pharmacy, Central South University, Changsha, China; ^3^Center for Pharmacogenetics, Department of Pharmaceutical Sciences, School of Pharmacy, University of Pittsburgh, Pittsburgh, PA, United States; ^4^Department of Pharmaceutics, School of Pharmacy, Ningxia Medical University, Yinchuan, China; ^5^Guangdong Second Provincial General Hospital, Guangzhou, China; ^6^School of Pharmaceutical Sciences, Guangzhou University of Chinese Medicine, Guangzhou, China

**Keywords:** ibuprofen, nanomicellar, prodrug, carrier, doxorubicin, co-delivery

## Abstract

Ibuprofen (IBU) is a non-steroidal anti-inflammatory drug (NSAID), which is widely used to reduce fever and treat inflammation and acute pain. Recently, its application in cancer treatment is also being explored. In this work, we synthesized a well-defined IBU-based amphiphilic diblock copolymer via reversible addition fragmentation transfer (RAFT) polymerization of IBU-based vinyl monomer. The amphiphilic copolymer POEG-*b*-PVBIBU (denoted as POVI) was composed of a hydrophilic poly(oligo(ethylene glycol)) block and a hydrophobic IBU-bearing prodrug block, which was able to self-assemble into prodrug nanomicelles. In addition, it could serve as a carrier to co-load other drugs including doxorubicin (DOX), paclitaxel (PTX), and docetaxel (DTX). By using DOX as a model anti-cancer drug, the delivery function of POVI carrier, including the drug release, *in vitro* cytotoxicity, cellular uptake, and *in vivo* antitumor activity, was evaluated. DOX-loaded POVI micelles exhibited sustained release of DOX. Besides, DOX/POVI micelles were effectively taken up by tumor cells with an efficiency comparable to that of free DOX. Moreover, *in vivo* studies showed that POVI carrier itself had modest antitumor activity. After loading DOX, the antitumor activity was significantly increased, which was significantly higher than that of free DOX. Our results suggest that POVI polymer represents a simple and effective dual-functional carrier for co-delivery of IBU and DOX to improve the anticancer activity.

## Introduction

The clinical applications of many antitumor drugs, such as doxorubicin (DOX), paclitaxel (PTX), and docetaxel (DTX), were limited by their low water solubility, poor bioavailability, and high toxic side effects (Lee et al., 2011; Senevirathne et al., 2016). To overcome these problems, various nanocarriers including micelles, dendrimers, and liposomes have been designed and developed for delivery of these hydrophobic antitumor drugs (Cho et al., 2008; Shen et al., 2010; Wei et al., 2013; Koudelka et al., 2015; Wang et al., 2015, 2017; Miao et al., 2017). Due to the small size, good stability, and unique core–shell structure, polymeric micelles have gained tremendous interest in the drug delivery field (Gaucher et al., 2005; Rösler et al., 2012). The hydrophobic core of micelles can encapsulate hydrophobic antitumor drugs via hydrophobic interactions, and the hydrophilic shell endows the micelles colloidal stability and protects the loaded drug from premature burst release.

It is well known that the constructing material, especially the hydrophobic domain of the polymeric micelles, plays a very important role in the delivery function of carriers (Zhang et al., 2014). Recently, increasing attention has been focused on the usage of bioactive compounds as hydrophobic moieties of polymeric carriers. These carriers themselves possess biological activity, which are termed as prodrug carriers (Craparo et al., 2013; Chen et al., 2016; Sun et al., 2017a). More importantly, some bioactive compounds with special structures in the prodrug carriers could interact with the co-loaded drug via π–π stacking effect, thereby improving the drug loading capacity (DLC; Sun et al., 2017b).

Non-steroidal anti-inflammatory drugs (NSAIDs), such as ibuprofen (IBU), indomethacin, and aspirin, are widely used to treat fever, arthritis, and rheumatic diseases. The anti-inflammatory effect of NSAIDs comes from the inhibition of the cyclooxygenase (COX) enzymes, including COX-1 and COX-2, which leads to the decrease of the production of prostaglandin, an important signaling molecule in the inflammation (Lim et al., 1999). Recently, accumulating evidences demonstrate that NSAIDs not only have anti-inflammatory effect, but also hold great potential in the prevention and treatment of several types of cancers (Ulrich et al., 2006). It has been suggested that the antitumor activity of NSAIDs can be partially explained by COX inhibition of prostaglandin synthesis (Ahnen, 1998). Other COX-independent mechanisms including inhibition of cell cycle progression (Gately and Kerbel, 2003) and induction of apoptosis (Davies et al., 2002) also contribute to the antitumor activity of NSAIDs. IBU is a commonly used NSAID that has been proven to inhibit proliferation of many tumor cells (Dial et al., 2006; Bonelli et al., 2011). Studies also demonstrate that IBU exhibited superior antitumor effect compared to other NSAIDs, mainly through the alteration of cell-cycle and induction of apoptosis (Andrews et al., 2002; Janssen et al., 2008). Moreover, compared to conventional anticancer drugs, IBU shows decreased side effect. Due to the potential of IBU in cancer treatment, IBU-conjugated prodrug polymers with various molecular structures have been developed to improve the water solubility and bioavailability of IBU. For example, Hasegawa and colleagues synthesized a series of amphiphilic diblock copolymers (PEG-PIBU) containing a hydrophilic poly(ethylene glycol) block and a hydrophobic IBU-bearing prodrug block (Hasegawa et al., 2013). By adjusting the length of hydrophobic IBU block, these polymers could self-assemble to form micelles with different morphologies. Wang et al. (2014) prepared another amphiphilic micellar carrier based on IBU-conjugated polymer for delivery of IBU. They found that in addition to the chemical conjugation, IBU could also be physically encapsulated into the micelles.

Although NSAIDs have potential antitumor activity, very high dosages of NSAIDs are needed to achieve a modest antitumor effect (Smalley and DuBois, 1997). Combination treatment of NSAIDs with other chemotherapeutic drugs is still necessary for cancer therapy (Endo et al., 2014; Lee et al., 2016). Recently, our group prepared a nanomicellar carrier that was self-assembled from PEG-Fmoc-IBU conjugate for co-delivery of IBU and PTX (Zhao et al., 2016). The IBU-based carrier showed synergistic antitumor effect with PTX, but there is only one IBU unit per carrier molecule, and thereby, large amounts of carriers were needed to deliver enough IBU dosage to tumor tissues, which might cause the unfavorable side effects.

Thus, in this work, we developed another IBU-containing polymeric carrier system with increased number of IBU units per polymer molecule via a simple synthesis method. We synthesized a POEG-b-PVBIbu diblock copolymer with a hydrophilic POEG block and a hydrophobic IBU block by reversible addition fragmentation transfer (RAFT) polymerization. The IBU-based prodrug polymer can form stable micelles with multiple IBU hydrophobic moieties in the core, which increases the solubility of IBU and allows more IBU being delivered into the tumor. Additionally, the micelle can serve as a carrier to encapsulate other hydrophobic chemotherapeutic drugs including DOX, DTX, and PTX. The size distribution and morphologies of drug-loaded micelles were evaluated. By using DOX as a model drug, the drug release, cellular uptake and antitumor activity were investigated *in vitro* and *in vivo*.

## Materials and Methods

### Materials

Vinylbenzyl chloride, 2, 2-Azobis(isobutyronitrile) (AIBN), 1,4-dioxane, trypsin-EDTA solution, 3-(4,5-dimethylthiazol-2-yl)-2,5-diphenyl tetrazolium bromide (MTT), and Dulbecco’s Modified Eagle’s Medium (DMEM) were all purchased from Sigma-Aldrich (St. Louis, MO, United States). AIBN was purified by recrystallization in anhydrous ethanol. DOX⋅HCl and DTX were purchased from LC Laboratories (Woburn, MA, United States). PTX was purchased from AK Scientific Inc. (Union City, CA, United States). Fetal bovine serum (FBS) and penicillin-streptomycin solution were all purchased from Invitrogen (NY, United States). POEG macroCTA were prepared as previously reported (Sun et al., 2016a).

### Synthesis of Ibu-Monomer

Vinylbenzyl chloride (1 eq.), IBU (1.5 eq.), and K_2_CO_3_ (2 eq.) were dissolved in DMF (5 eq.). The mixture was stirred at 50°C for 24 h, and then equal volume of water was added. The crude product was extracted by DCM for three times, and then purified by silica gel column chromatography.

### Synthesis of POEG-b-PVBIbu Polymers

Ibu-monomer (180 mg, 0.559 mmol), POEG macroCTA (280 mg, 0.0372 mmol), AIBN (2 mg, 0.0124 mmol), and 2 mL dried 1, 4-dioxane were placed in a Schlenk tube. The mixture was degassed under N_2_ by three freeze-pump-thaw cycles, and then immersed into an oil bath at 90°C. After 24 h, the polymerization was quenched by placing the Schlenk tube into liquid nitrogen. The polymer mixture was precipitated in petroleum ether for three times and dried in vacuum.

### Characterization of the Synthesized Monomer and Polymers

^1^H NMR spectrum was conducted on a Varian 400 FT-NMR spectrometer at 400.0 MHz with CDCl_3_ as the solvent. Molecular weight (*M*_n_ and *M*_w_) and polydispersity index (*M*_w_/*M*_n_) of the synthesized polymers were determined by gel permeation chromatography (GPC) equipped with a Waters 2414 refractive index detector, a Waters 515 HPLC pump, and a Waters 717 Plus Autosampler. THF was used as the eluent with a flowing rate of 1.0 mL/min at 35°C. A series of polystyrene standards with narrow molecular weight distribution were used for calibration.

### Preparation of Drug-Free Micelle and Drug-Loaded Micelles

The blank POVI micelles and drug-loaded micelles were prepared by dialysis method. The polymer POVI and drug (DOX, PTX, or DTX) with certain mass ratio were dissolved in DMSO, which were then transferred to dialysis bags (MWCO 3.5 kDa), and dialyzed against PBS for 24 h. The size distributions and morphologies of blank and drug-loaded micelles were measured by dynamic light scattering (DLS) and transmission election microscopy with negative staining.

Doxorubicin concentration was detected by Fluorescence Microplate Reader with excitation wavelength of 490 nm and emission wavelength of 590 nm. PTX concentration was measured by reverse phase high-performance liquid chromatography (RP-HPLC) with a mobile phase of methanol/water (70:30 v/v) at the flow rate of 1.0 mL/min, and UV detection at 227 nm. DTX concentration was detected by RP-HPLC with a mobile phase of acetonitrile/water (50:50 v/v) at the flow rate of 1.0 mL/min, and UV detection at 230 nm.

Drug loading capacity and drug loading efficiency (DLE) were calculated according to the following formula:

DLC (%) = [weight of drug loaded/(weight of polymer + drug used)] × 100%

DLE (%) = (weight of loaded drug/weight of input drug) × 100%.

### Critical Micelle Concentration (CMC) of POVI Micelle

Critical micelle concentration (CMC) of POVI micelle was measured using Nile red as a fluorescence probe (Klaikherd et al., 2009; Sun et al., 2016b); 10 μL Nile red in chloroform was added into each tube, and the solvent was removed by air flow and vacuum pump. Polymer solution (120 μL) with concentrations ranging from 1 × 10^-4^ to 0.5 mg/mL was added to each tube, and incubated overnight. The fluorescence intensity of each sample was detected by a Synergy H1 Hybrid Multi-Mode Microplate Reader (Winooski, VT, United States) at a wavelength of 480/620 nm (excitation/emission).

### *In Vitro* Drug Release Study

Doxorubicin-loaded POVI micelles (0.5 mg DOX/mL) in PBS (pH = 7.4) was placed in a dialysis bag (MWCO = 12 kDa, Spectrum Laboratories), and incubated in a 200-mL beaker with PBS containing 0.5% (w/v) Tween 80, with gentle shaking (100 rpm/min) at 37°C. DOX solution in saline with the same concentration was used as a control. The concentration of DOX outside the dialysis bag was measured by a fluorescence microplate reader at designated time points and the values were reported as the means of triplicate samples.

### Cell Culture

Mouse metastatic breast cancer cell line 4T1.2, human breast cancer cell line MCF-7, and androgen-independent human prostate cancer cell line PC-3 were cultured at 37°C in DMEM containing 10% FBS and 1% penicillin-streptomycin in a humidified environment with 5% CO_2_.

### *In Vitro* Cytotoxicity Study

4T1.2 (1500 cells/well), MCF-7 (4000 cells/well), or PC-3 (2500 cells/well) were seeded in 96-well plates and incubated for 24 h. Then the cells were treated with various concentrations of drug-free POVI micelles, DOX-loaded POVI micelles, or DOX. After incubation for 72 h, 20 μL of MTT in PBS (5 mg/mL) was added into each well and further incubated for 4 h. The medium was then removed, and DMSO was added to solubilize the MTT formazan. The absorbance of each well was measured with a microplate reader at a wavelength of 550 nm and a reference wavelength of 630 nm. Untreated cells were used as a control. Cell viability was calculated as [(OD_treat_ - OD_blank_)/(OD_control_ - OD_blank_) × 100%].

### Intracellular Trafficking

4T1.2 cells (15,000/well) were seeded in glass bottom dishes (In Vitro Scientific, United States), and incubated overnight. The cells were treated with free DOX and DOX/POVI micelles (DOX concentration: 15.5 μg/mL) for 2 and 4 h separately. Then cells were stained with Hoechst 3342 for 15 min, and washed with cool PBS for three times. The intracellular distributions of different DOX formulations were observed under a confocal laser scanning microscope (CLSM, FluoView 1000, Olympus, Japan).

### Animals

Female BALB/c mice (6–8 weeks) were purchased from Charles River (Davis, CA, United States). All animals were housed under pathogen-free conditions according to Association for Assessment and Accreditation of Laboratory Animal Care (AAALAC) guidelines. All animal-related experiments were performed in full compliance with institutional guidelines and approved by the Animal Use and Care Administrative Advisory Committee at the University of Pittsburgh.

### *In Vivo* Therapeutic Study

A syngeneic murine breast cancer model (4T1.2) was used to evaluate the therapeutic efficacy of DOX-loaded POVI micelles. 4T1.2 cells (2 × 10^5^ cells/mouse) were inoculated s.c. at the right flank of female BALB/c mice. When the tumor volume reached ∼50 mm^3^ (day 0), mice were randomly divided into four groups (*n* = 3) and received i.v. administration of saline (control), POVI micelles, free DOX, and DOX-loaded POVI micelles, respectively, on days 0, 3, 6, 9, 12, 15, and 18. The DOX dosage for free DOX and DOX-loaded POVI micelles was 5 mg DOX/kg. The dosage for POVI micelles was 73 mg POVI/kg, which was the same as that of POVI in DOX-loaded POVI micelles. Tumor volumes were measured with digital caliper and calculated as *V* = (*L* × *W*^2^)/2, where *L* is the longest and *W* is the shortest tumor diameters (mm). Each group was compared by relative tumor volume (RTV = *V*/*V*_0_, *V*_0_ was the tumor volume prior to first treatment). Mice were sacrificed when the tumor volume reached ∼2000 mm^3^. The size and weight of tumor stripped from the mice were measured. To evaluate the potential toxicity, the body weights were also monitored during the entire course of treatment.

### Histochemical Staining

After *in vivo* therapeutic study, tumor tissues were excised and preserved in 4% formaldehyde in PBS, followed by embedment in paraffin. The paraffin-embedded tumor samples were cut into thin slices of 5 μm with an HM 325 Rotary Microtome. Then the slices were stained with hematoxylin and eosin (H&E) for histopathological examination under a Zeiss Axiostar plus Microscope (PA, United States).

### Statistical Analysis

All results were reported as the mean ± SD unless otherwise indicated. Statistical analysis was performed with Student’s *t*-test for two groups, and one-way ANOVA for multiple groups, followed by Newman–Keuls test if *P* < 0.05. In all statistical analysis, *P* < 0.05 was considered statistically significant.

## Results

### Synthesis of POEG-b-PVBIbu Polymers

Reversible addition fragmentation transfer polymerization of functional monomer has become an attractive strategy to obtain well-defined functional polymers for drug/gene delivery (Sun et al., 2013a,b; Tucker et al., 2015). In this work, we synthesized a well-defined IBU-based prodrug polymer via RAFT polymerization of IBU-conjugated monomer, and investigated its function as a dual-functional carrier for co-delivery of other chemotherapeutic drugs.

**SCHEME 1:**
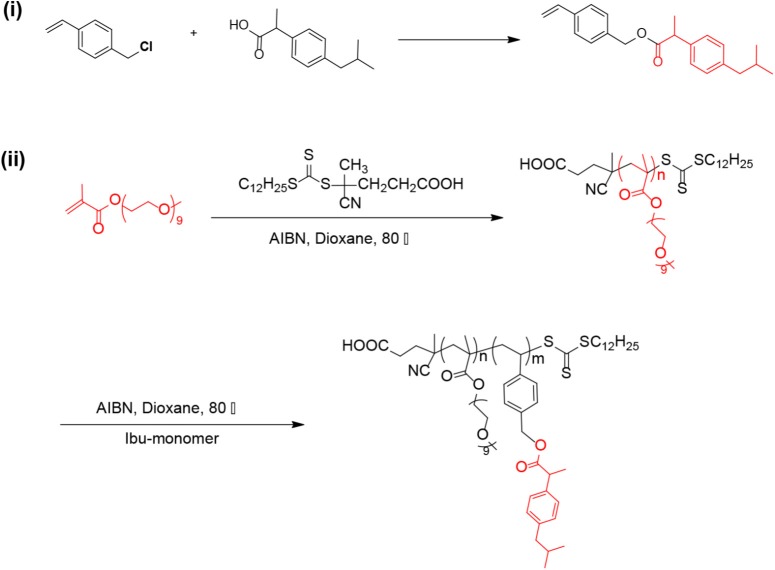
Synthesis of the IBU-monomer and POEG-*b*-PVBIBU (POVI) polymers via RAFT polymerization.

**FIGURE 1:**
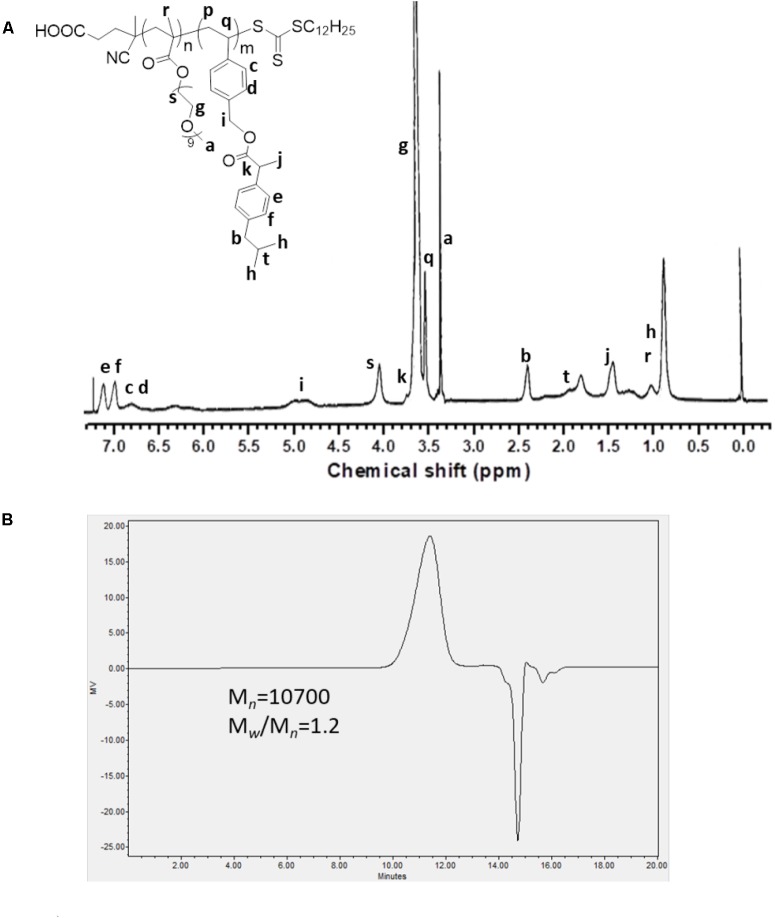
**(A)** ^1^H NMR spectrum of the POEG-*b*-PVBIBU (POVI) diblock copolymer in CDCl_3_. **(B)** Gel permeation chromatography curve of the POVI diblock polymer.

As shown in **Scheme 1**, we first designed and synthesized a vinylbenzyl derivative of IBU (IBU-monomer) where IBU was conjugated with vinylbenzyl chloride via a hydrolyzable ester linkage. Then, the macro-chain transfer agent POEG macroCTA was synthesized by RAFT polymerization as previously reported (Sun et al., 2016a), which further initiated the polymerization of IBU-monomer to give the POEG-*b*-PVBIbu block copolymers. The structures of the monomer and polymer were confirmed by ^1^H NMR (**Figure 1**). As shown in **Figure 1A**, the average degree of polymerization (DP) of the Ibu-monomer was determined to be 12 by comparing the intensities of *I*_b_ and *I*_a_. GPC showed unimodal peaks for the polymer with number-average molecular weight *M*_n_ of 10,700, and polydispersity of 1.20 (**Figure 1B**), which indicated the successful synthesis of the well-defined POEG-*b*-PVBIbu (POVI) block copolymers.

### Preparation and Physicochemical Characterization of POVF Blank Micelles and Drug-Loaded Micelles

**FIGURE 2:**
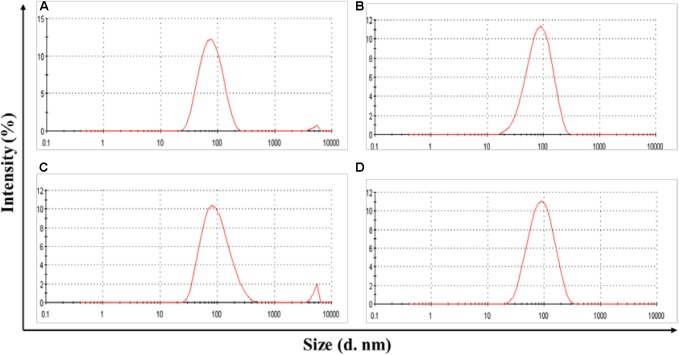
Size distribution of POVI **(A)**, DOX/POVI **(B)** (mass ratio of micelle to DOX = 10:1), PTX/POVI **(C)**, and DTX/POVI micelles **(D)** (mass ratio of micelle to PTX/DTX = 20:1).

**FIGURE 3:**
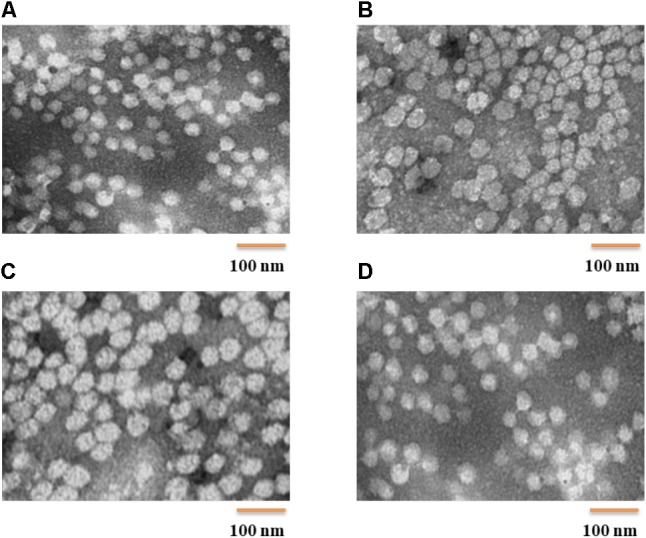
TEM images (direct mag: 150,000X) of POVI **(A)**, DOX/POVI **(B)** PTX/POVI **(C)**, and DTX/POVI **(D)** micelles.

POVF blank micelles and drug-loaded micelles (including DOX/POVI, PTX/POVI, and DTX/POVI) were successfully prepared by dialysis method, and the physicochemical characterization is summarized in **Table 1**. DLS analysis showed that POVF polymers could form nano-sized micelles with a diameter of 82 nm. The sizes of micelles slightly increased to 92, 106, and 98 nm after loading of DOX, PTX, and DTX, respectively (**Figure 2**), which may be due to that the encapsulated hydrophobic drugs enlarged hydrophobic core of the micelles. The morphologies of the blank and drug-loaded micelles were evaluated by transmission electron microscopy (TEM). As shown in **Figure 3**, these micelles are spherical in morphology with uniform size. DLE and capacity were measured by a fluorescence microplate reader. As shown in the **Table 1**, POVI polymer was able to load DOX with a loading capacity of 6.4%. In addition, POVI polymers could also serve as a carrier to load other drugs such as PTX and DTX.

**Table 1:** Physicochemical characterization of POVI blank micelle and drug-loaded micelles.

Micelles	Size (nm)^b^	PDI^c^	Carrier: DOX mass ratio	DLE%^d^	DLC%^d^
POVI^a^	82.35	0.207	–	–	–
DOX/POVI	92.78	0.194	10: 1	69.98%	6.4%
PTX/POVI	106.7	0.281	20: 1	72.90%	3.5%
DTX/POVI	98.30	0.204	20: 1	41.74%	2.0%


*^*a*^Concentration of blank micelle was 20 mg/mL. ^*b*^Measured by DLS. ^*c*^PDI, polydispersity index; ^*d*^DLC%, drug loading capacity; DLE%, drug loading efficiency.*

**FIGURE 4:**
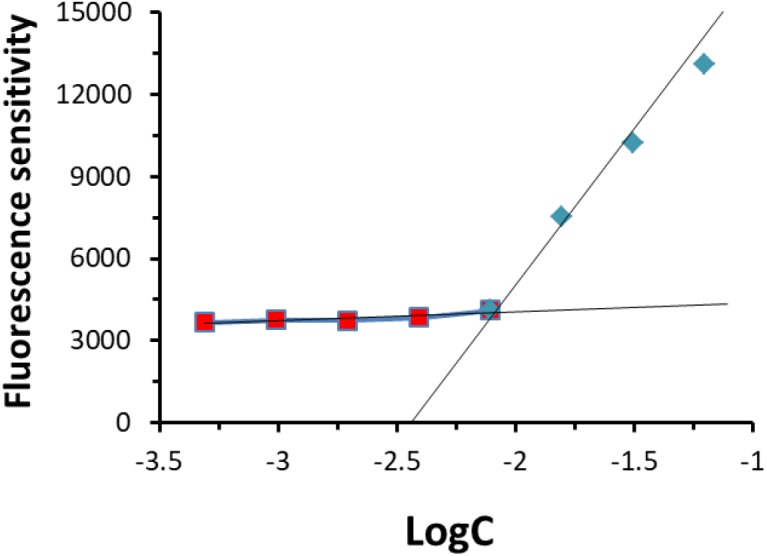
Critical micelle concentration (CMC) of POVI micelle.

The CMC value of POVF micelles was evaluated with Nile red as a fluorescence probe. As shown in **Figure 4**, the CMC of POVI micelles was measured to be 9.8 mg/L. The low CMC suggests a good stability of our micelles following dilution in the blood after i.v. administration.

### Release Kinetics of DOX

**FIGURE 5:**
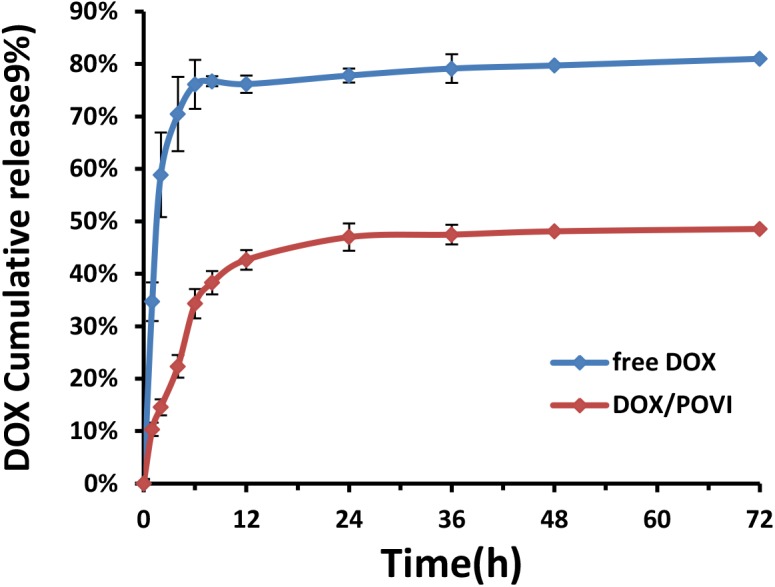
DOX release from DOX-loaded POVI micelles via a dialysis method. PBS solution was used as release medium, and DOX concentrations were analyzed at 0, 1, 2, 4, 6, 8, 12, 24, 36, 48, and 72 h.

The release kinetics of DOX from DOX/POVI micelles was evaluated using a dialysis method, and free DOX solution was used as a control. As shown in **Figure 5**, free DOX solution showed a burst DOX release of almost 80% within the first 12 h, while DOX/POVI micelles showed a slow DOX release of about 43%. Even after 72 h, less than 50% of DOX was released from DOX/POVI micelles.

### *In Vitro* Cytotoxicity of DOX/POVI

**FIGURE 6:**
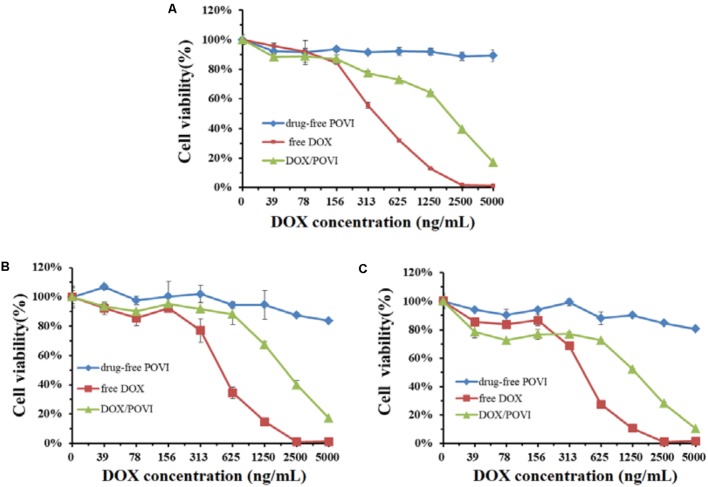
*In vitro* cytotoxicity of DOX/POVI micelles. 4T1.2 **(A)**, MCF7 **(B)**, and PC3 **(C)** cells were treated with DOX-free POVI, DOX/POVI micelles, and free DOX for 72 h, and the cytotoxicity was determined by MTT assay, respectively.

*In vitro* cytotoxicity of DOX/POVI micelles was evaluated in 4T1.2, MCF7, and PC3 cell lines. As shown in **Figure 6**, POVI carrier did not show any remarkable cytotoxicity among all cell lines tested. DOX/POVI micelles and DOX solution exhibited potent cytotoxicity in a concentration-dependent manner. DOX/POVI micelles exhibited lower cytotoxicity than free DOX in the three cell lines, which is likely due to the slow release of DOX from DOX/POVI micelles.

### Intracellular Trafficking

**FIGURE 7:**
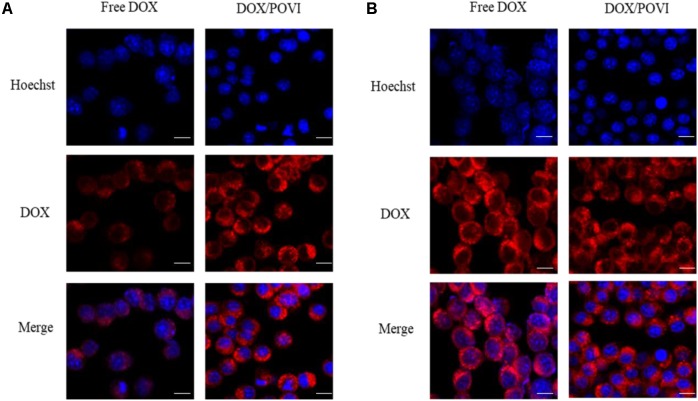
Intracellular trafficking of DOX in 4T1. Cells were treated with free DOX, DOX/POVI micelle at 15.5 μg/mL for 2 h **(A)** and 4 h **(B)**. The nuclei were stained with Hoechst 33342. Scale bar: 20 μm.

Intracellular trafficking and distribution of DOX/POVF micelles were studied by confocal laser scanning microcopy (CLSM). 4T1.2 cells were incubated with free DOX and DOX/POVF micelles for 2 and 4 h, respectively. At 2 h, cells treated with DOX/POVF micelles exhibited strong DOX fluorescence signals around the nucleus, similar to the cells treated with free DOX (**Figure 7A**). At 4 h, increased signals of red fluorescence appeared in nuclei, both for DOX/POVF micelle group and free DOX group (**Figure 7B**).

### *In Vivo* Therapeutic Efficacy Evaluation

**FIGURE 8:**
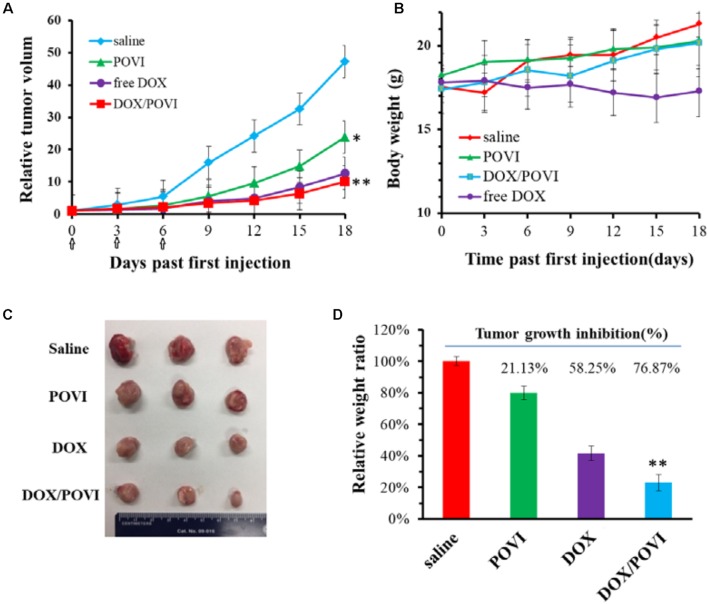
*In vivo* therapeutic efficacy of DOX/POVI micelles. BALB/c mice in 4T1.2 murine breast cancer model were injected with saline, POVI, free DOX, and DOX/POVI (5 mg DOX/kg body weight) on days 0, 3, and 6, separately. ^∗^*P* < 0.05; ^∗∗^*P* < 0.01 (vs saline). **(A)** Relative tumor volume curves of mice receiving different treatments. **(B)** Changes of body weight in mice were monitored. **(C)** Image of tumors stripped from mice on the day 18. **(D)** Tumor weights of mice were measured on day 21 and tumor growth inhibition (%) was calculated. ^∗∗^*P* < 0.05 (vs free DOX).

The *in vivo* therapeutic efficacy of DOX/POVI micelles was evaluated in the 4T1.2 breast tumor-bearing BALB/c mice. The mice were treated with saline, POVI carrier, free DOX, and DOX/POVI micelles by i.v. injection, respectively. As shown in **Figure 8A**, DOX exhibited a modest tumor growth inhibition at a dose of 5 mg DOX/kg. DOX/POVI micelles were more effective than DOX in inhibiting tumor growth (*p* < 0.05) at the same dose. Interestingly, POVI carrier alone also showed tumor growth inhibition effect, although it was less effective than DOX.

No significant changes were found in the body weights of mice following various treatments (**Figure 8B**), indicating that all treatments were well tolerated. **Figure 8C** shows the photographs of tumors removed from the mice after various treatments. Compared to saline treatment group, DOX/POVI treatment led to an obvious decrease in tumor size. The tumor growth inhibition rate of DOX/POVI micelles is calculated to be significantly higher than those of free DOX and POVI blank micelles (*P* < 0.05; **Figure 8D**).

**FIGURE 9:**
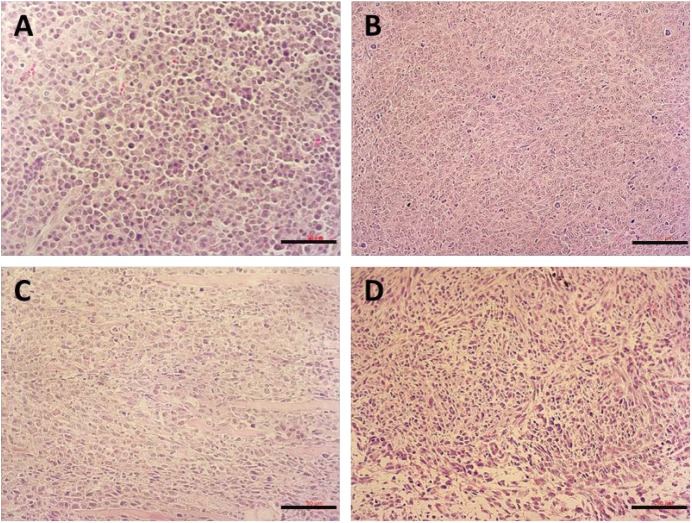
H&E stained tumor tissues from mice received different treatments: **(A)** saline, **(B)** POVI, **(C)** free DOX, and **(D)** DOX/POVI at a dosage of 5 mg DOX/kg. Three injections were made into BALB/c mice bearing 4T1.2 tumor on days 0, 3, and 6, separately. Tumor tissues were harvested on day 18. Scale bar: 50 μm.

**Figure 9** shows the images of H&E-stained slices of tumors collected after the completion of *in vivo* therapy study. Tumors treated with saline showed normal tumor morphology with large nuclei. In contrast, tumors in other treatment groups showed shrunk nuclei. Among them, DOX/POVF treatment resulted in the most significant tumor necrosis.

## Discussion

Ibuprofen is one type of NSAID that not only possesses anti-inflammatory effect, but also demonstrates great potential in inhibiting proliferation of various types of tumor cells (Dial et al., 2006; Bonelli et al., 2011). Currently, combination of IBU with chemotherapeutic drugs is being evaluated as a new regimen for clinical cancer treatment. However, free drugs are readily eliminated in the blood and they lack tumor targeting. Thus, co-delivery of IBU and a chemotherapeutic drug into tumors using a delivery vehicle is highly needed. Previously, we reported a nanomicellar carrier based on PEG-Fmoc-IBU conjugate for co-delivery of IBU and PTX (Zhao et al., 2016). Although PEG-Fmoc-IBU carrier was effective in loading and delivering PTX into tumors, the efficiency of delivery of IBU itself was relatively low because there was only one IBU unit per polymer molecule. In the present work, we developed a new IBU-conjugated polymeric carrier POVI with increased units of IBU per carrier molecule via a facile RAFT polymerization method. Compared to the previous system, the IBU loading capacity in the new POVI system was increased from 8.2 to 20.9%, indicating that more IBU could be delivered into the tumor tissues when injecting the same amount of carriers.

First, we designed and synthesized a novel IBU-conjugated vinylbenzyl monomer, which was further polymerized to yield the IBU-containing block copolymers. Compared with the post-conjugation method in which a drug was conjugated to the polymerized backbone, our method has an obvious advantage of simplicity. In addition, it avoids the low drug conjugation efficiency caused by the steric hindrance.

The synthesized POVI polymer could serve as a carrier that is effective in loading various types of chemotherapeutic drugs, including DOX, PTX, and DTX. We found that the monomer structure is critical for the loading capacity of the carrier. In the preliminary study, we also synthesized another IBU-based polymer using IBU-conjugated hydroxyethyl methacrylate monomer. However, the polymer obtained performed poorly in encapsulating other chemotherapeutic drugs (data not shown). When we introduced vinylbenzyl group into the polymers, the DLC was significantly improved, suggesting that, in addition to hydrophobic interaction, the π–π stacking effect between carrier and the chemotherapeutic drug may also contribute to the overall DLC. Moreover, the structures of chemotherapeutic drugs also affected the loading capacity. The POVI carrier was more effective in encapsulating DOX compared to other chemotherapeutic drugs, which might be due to the stronger carrier/DOX interaction.

The DOX-loaded micelles showed small size with a diameter of ∼90 nm (**Figure 2**), which was optimal for a long blood circulation time and passive targeting to solid tumor sites through the enhanced permeability and retention (EPR) effect (Mitra et al., 2001; Meng et al., 2011; Kobayashi et al., 2014). DOX-loaded micelles showed slow and sustained drug release (**Figure 5**), which could prevent the premature release of encapsulated DOX before entering into tumors. The slow release of DOX from the micelles may be due to the strong interaction (e.g., hydrophobic interaction and π–π stacking effect) between DOX and POVI carrier (Sun et al., 2016a).

*In vitro* cytotoxicity showed that DOX formulated in the carrier was less effective in inhibiting the proliferation of tumor cells compared to free DOX. The *in vitro* cytotoxicity comes from the overall outcome of intracellular uptake and drug release. The DOX/POVI micelles showed similar cellular uptake compared to free DOX (**Figure 7**); the lower *in vitro* cytotoxicity of DOX/POVI micelles might be attributed to the slow release of DOX from the micelles.

POVI carrier was not effective in inhibiting the proliferation of cultured tumor cells *in vitro* (**Figure 6**) at the concentrations that were used for DOX delivery *in vitro*. This may be due to the slow release of IBU during a short period of culture and its relatively low potency. However, *in vivo* data showed that the POVI carrier itself showed moderate antitumor activity (**Figure 8**). In our previous study, PEG-IBU conjugate could also inhibit tumor growth compared with control group (Zhao et al., 2016). So the antitumor activity of POVI carrier *in vivo* may come from the released IBU mediated by various kinds of enzymes in the tumor tissues over a relatively long period of time. Moreover, DOX-loaded micelles showed a much more pronounced antitumor activity compared with free DOX, which is different from the *in vitro* cytotoxicity assay. The improved therapeutic efficacy of DOX/POVI micelles is likely attributed to the combination effect of the carrier with co-delivered DOX. The improved delivery of DOX by the POVI carrier may also play a role. More studies are warranted to further investigate the underlying mechanisms.

## Conclusion

We have developed a new IBU-based prodrug di-block polymer POVI via facile RAFT polymerization. POVI polymer could load various hydrophobic drugs including DOX, PTX, and DTX with high loading capacity. DOX/POVI micelles showed similar cytotoxicity and cellular uptake compared to free DOX. More importantly, DOX/POVI micelles were more effective in inhibiting the tumor growth than free DOX *in vivo*. Our results suggest that POVI polymer can be employed as a safe and effective dual-functional carrier for co-delivery of other chemotherapeutic drugs.

## Author Contributions

SL, JS, and YH conceived and designed the study. JS and ZL contributed to chemical synthesis and micelle characterization. ZL, YL, JX, YC, LL, JL, and QL contributed to biological study. ZL, JS, YL, and JX analyzed the data. ZL, JS, and SL drafted the manuscript. All authors approved the final version of the manuscript.

## Conflict of Interest Statement

The authors declare that the research was conducted in the absence of any commercial or financial relationships that could be construed as a potential conflict of interest.
